# Urinary succinate is associated with kidney injury in type 2 diabetes mellitus: a cross-sectional analysis

**DOI:** 10.3389/fendo.2026.1908195

**Published:** 2026-10-26

**Authors:** Ting Cai, Yihan Jiao, Xinyu Chen, Xinyue Kong, Caimei Chen, Hanzhi Chen, Yue Zhang, Jia Hua, Xiaobin Liu, Bin Cai, Xiaowei Zhu, Liang Wang

**Affiliations:** 1 The Affiliated Wuxi People's Hospital of Nanjing Medical University, Wuxi People's Hospital, Wuxi Medical Center, Nanjing Medical University, Wuxi, China; 2 Department of Anorectal Surgery, Wuxi Affiliated Hospital of Nanjing University of Chinese Medicine, Wuxi, China

**Keywords:** biomarker, diabetic kidney disease, kidney injury, type 2 diabetes mellitus, urinary succinate

## Abstract

**Objective:**

Diabetic kidney disease (DKD) is a common microvascular complication in patients with type 2 diabetes mellitus (T2DM), yet reliable biomarkers for disease assessment and effective risk stratification remain limited. Succinate is a signaling molecule implicated in kidney diseases. This study aimed to investigate the association between urinary succinate and kidney injury in patients with T2DM.

**Methods:**

Cross-sectional study of 240 participants: healthy controls (n = 25), patients with non-DKD (n =40), and 175 patients with T2DM (85 without DKD and 90 with DKD). Urinary succinate levels were measured using a colorimetric assay kit.

**Results:**

Urinary succinate levels were significantly elevated in patients with T2DM but not in patients with non-DKD, with the highest levels observed in patients with DKD. Urinary succinate correlated negatively with estimated glomerular filtration rate (eGFR) and positively with serum creatinine, urinary albumin-to-creatinine ratio (UACR), urinary N-acetyl-β-D-glucosaminidase (NAG), and urinary neutrophil gelatinase-associated lipocalin (NGAL) in patients with DKD, but only correlated with urinary NAG in patients with T2DM without DKD. In multivariable liner regression analysis, urinary succinate was found to be independently associated with eGFR in patients with DKD after adjustment for age, male sex, smoking history, hypertension, HbA1c > 7.0%, uric acid, UACR, and the use of renin-angiotensin system inhibitors (RASi) and sodium-glucose co-transporter 2 inhibitors (SGLT2i). Receiver operating characteristic (ROC) analysis for detecting eGFR < 60 mL/min/1.73m^2^ in DKD yielded an AUC of 0.70, with a sensitivity of 57.14% and a specificity of 75.00%.

**Conclusion:**

Urinary succinate levels are elevated in patients with T2DM, particularly in DKD, but not increased in patients with non-DKD. Urinary succinate is independently associated with eGFR, and shows moderate diagnostic performance for renal insufficiency in DKD. These findings suggest that urinary succinate may serve as a potential non-invasive biomarker for diabetes-related kidney injury. Prospective studies are needed to evaluate its predictive value for early detection and disease progression of DKD.

## 1 Introduction

Diabetic kidney disease (DKD) is a common microvascular complication in patients with type 2 diabetes mellitus (T2DM), characterized by increased albuminuria and progressive renal function decline. Despite recent therapeutic advances having modestly slowed disease progression, they fail to halt the relentless deterioration of renal function in patients with DKD (1, 2), owing to its multifactorial pathogenesis. Consequently, DKD has emerged as the leading cause of end-stage renal disease (ESRD) (3, 4). Currently, the diagnosis and monitoring of DKD rely mainly on urinary albumin-to-creatinine ratio (UACR) and estimated glomerular filtration rate (eGFR) (5). However, not all patients with DKD who have microalbuminuria progress to macroalbuminuria (6). Moreover, approximately 20% of patients with diabetes exhibit renal function deterioration even in the absence of significant albuminuria (7). Given the complexity of the pathogenesis of DKD and diversity of its clinical manifestations, UACR, as an indicator of glomerular injury, has limited value in the diagnosis and risk stratification of the disease. Furthermore, eGFR has suboptimal sensitivity for the detection of early kidney injury in DKD. Consequently, there is an urgent need for new biomarkers to facilitate the disease assessment and effective risk stratification of DKD.

DKD is known to arise from diabetes-driven metabolic disorders that contribute to progressive renal function impairment (8). Accumulating human metabolomic studies have highlighted disturbances in tricarboxylic acid (TCA)-cycle-derived metabolites as important features of diabetes-associated metabolic reprogramming (9, 10). Among these TCA-cycle intermediates, succinate is emerging as a metabolite of particular interest in diabetic kidney injury. Succinate, converted from succinyl-CoA via succinyl-CoA synthetase, was previously thought to participate exclusively in mitochondrial energy metabolism. Nonetheless, landmark studies have identified succinate as a signaling molecule acting independent of canonical metabolism in the pathogenesis of kidney diseases, including DKD (11–14). Toma et al. (14) found that hyperglycemia caused an increased succinate level in kidney sections of diabetic animals, activating the G protein-coupled receptor 91 (GPR91, also known as succinate receptor 1 (SUCNR1)) signaling pathway and promoting renin release, ultimately leading to hypertension, glomerular hyperfiltration and renal injury. Recent clinical evidence demonstrated that elevated levels of urinary succinate in patients with DKD were independently associated with albuminuria and DKD (15). However, the elevation of urinary succinate in diabetes, its correlation with tubular injury beyond glomerular damage, and its independent association with kidney injury severity in DKD remain incompletely elucidated.

In the current study, we addressed the above-mentioned questions by: (1) comparing urinary succinate levels across patients with diabetes, healthy controls and patients with non-diabetic kidney disease (non-DKD); (2) analyzing its correlations with kidney injury markers, including serum creatinine, eGFR, UACR, urinary N-acetyl-β-D-glucosaminidase (NAG) and urinary neutrophil gelatinase-associated lipocalin (NGAL); (3) assessing its independent association with eGFR, as well as its diagnostic performance for renal insufficiency (defined as an eGFR < 60 mL/min/1.73 m²) in patients with DKD.

## 2 Methods

### 2.1 Study population

The study was approved by the Institutional Ethics Committee of Wuxi People’s Hospital of Nanjing Medical University (Approval No. 2023KY23129). All participants provided written informed consent prior to study enrollment. A total of 240 participants were enrolled in this cross-sectional study from September 2023 to January 2025, and were divided into four groups: (1) the healthy control group (n = 25); (2) patients with non-DKD (non-DKD group, n = 40); (3) patients with T2DM but without DKD (T2DM−DKD group, n = 85); and (4) patients with DKD (T2DM+DKD group, n = 90). The healthy control group comprised individuals without diabetes mellitus, hypertension, inflammation, coronary artery disease, malignant neoplasm, or any known kidney disease. All individuals in this group exhibited normal eGFR and normal routine urinalysis. The non-DKD group comprised 14 cases of biopsy-proven IgA nephropathy, 11 cases of membranous nephropathy, 4 cases of biopsy-proven minimal change disease/focal segmental glomerulosclerosis, 4 cases of hypertensive nephropathy, and 7 cases of other conditions, all without a history of diabetes. Subjects who had received corticosteroids, immunosuppressants, or biologics within the past 6 months were excluded from the non-DKD group. DKD was defined as UACR ≥ 30 mg/g urinary creatinine (Ucr) and/or eGFR < 60 mL/min/1.73m^2^ for more than 3 months in patients with T2DM, after excluding other causes of kidney disease. The following criteria were used for subject exclusion: (1) subjects younger than 18 years of age; (2) subjects with diabetic ketoacidosis or diabetic hyperosmolar state; (3) subjects with severe liver insufficiency, defined as transaminase levels three times above the upper limit of normal range; (4) subjects receiving renal replacement therapy; (5) subjects with hypertension and uncontrolled blood pressure, defined as a systolic blood pressure of ≥ 180 mmHg and/or a diastolic blood pressure of ≥ 110 mmHg; (6) subjects with an untreated malignant neoplasm or uncontrolled active inflammatory disease.

### 2.2 Clinical information

Clinical data were collected from patient histories and detailed medical record review. Morning spot urine samples were submitted to the local laboratory for routine urinalysis and UACR measurement. A comprehensive biochemical blood test was performed, including hemoglobin A1c (HbA1c), hemoglobin (Hb), albumin, high-density lipoprotein cholesterol (HDL), low-density lipoprotein cholesterol (LDL), serum creatinine, urea and uric acid. eGFR was calculated with the CKD-EPI equation.

### 2.3 Examination of urinary succinate, NAG and NGAL

Morning spot urine samples were collected for biochemical analysis. The urine samples were centrifuged at 3,000 rpm at 4°C for 15 minutes, and aliquots were stored at -80 °C until further analysis. No clinical information was available during the measurement because urine samples were labeled only with identification numbers. Succinate levels in neat urine samples were measured by a colorimetric succinate assay kit (ab204718, Abcam) following the manufacturer’s protocol. This kit converts a colorless probe into a colored product based on enzymatic reaction, with absorbance read at 450 nm, and has a reported detection limit of < 40 μM. 36 randomly selected samples (15% of the total cohort) were measured in duplicate, and the mean intra-assay coefficient of variation was 1.42% (range: 0.02%–6.58%), demonstrating acceptable reproducibility. Urinary NAG levels were quantified using a colorimetric assay kit (A031-1-1, Nanjing Jiancheng Bioengineering Institute) according to the manufacturer’s protocol. Urinary NGAL levels were assessed using an enzyme-linked immunosorbent assay (ELISA) kit (ml064308, Shanghai Enzyme-linked Biotechnology Co.) following the manufacturer’s protocol. The urinary levels of succinate, NAG and NGAL were finally normalized to urinary creatinine.

### 2.4 Statistical analysis

The statistical analyses were performed using SPSS 28.0 and Prism 10.0 software packages. Continuous variables with normal distribution were presented as the mean ± standard deviation, and non-normally distributed variables were expressed as median with interquartile range. Urinary succinate was log_10_-transformed to improve normality. Categorical variables were expressed as counts (percentages). Comparisons between groups for normally distributed continuous variables were performed using an unpaired Student’s t-test or one-way ANOVA. Variables that did not meet the criteria for normal distribution were analyzed by a Mann-Whitney U-test or Kruskal-Wallis test. The chi-squared test was used for categorical variable comparison. The association between urinary succinate and UACR, urinary NAG, urinary NGAL, serum creatinine and eGFR was examined by Pearson’s correlation or Spearman’s correlation analysis according to data normality. Multivariable linear regression analysis was performed to evaluate the independent association between urinary succinate and eGFR in patients with DKD, adjusting for potential confounders. Covariates included in the multivariable liner regression models were selected based on established risk and protective factors for renal function in patients with DKD, informed by both published literature and current clinical guidelines (5, 16, 17). Model 1 was adjusted for age and sex as basic demographic confounders. Model 2 was further adjusted for additional risk factors, including smoking history, hypertension, HbA1c > 7.0%, uric acid, and UACR. In addition, the use of RASi and SGLT2i was included in Model 2, as these renoprotective guideline-recommended medications may affect the association between urinary succinate and eGFR. Receiver operating characteristic (ROC) analysis was performed to evaluate the diagnostic performance of urinary succinate for the detection of renal insufficiency in patients with DKD. A p-value of less than 0.05 was considered statistically significant. For missing data, two missing UACR values in the non-DKD group were imputed using the group median, and no missing values were detected for other variables in this study.

## 3 Results

### 3.1 Baseline Characteristics of the study population

The demographic information and clinical characteristics of the study population are summarized in **Table 1**. The median ages of the healthy controls, patient with non-DKD, patients with T2DM without DKD, and patients with DKD were 39.00 years, 53.50 years, 59.00 years and 60.00 years, respectively (*p* < 0.001). No significant sex differences were observed. Comparisons across the four groups revealed significant differences in smoking history, body mass index (BMI), prevalence of hypertension, hemoglobin (Hb), albumin, HDL, LDL, uric acid, urea, serum creatinine, eGFR, and the use of renin-angiotensin system inhibitors (RASi) and sodium-glucose co-transporter 2 inhibitors (SGLT2i) (all *p* < 0.05). UACR, urinary NAG, and urinary NGAL differed between patients with non-DKD, patients with T2DM without DKD, and patients with DKD (all *p* < 0.05). Notably, patients with DKD had a longer duration of T2DM and a higher prevalence of diabetic retinopathy than patients with T2DM without DKD (both *p* < 0.001).

**Table 1:** Characteristics of study population.

Characteristics	Healthy control (n = 25)	Non-DKD (n = 40)	T2DM−DKD (n = 85)	T2DM+DKD (n = 90)	*p*
Age (years)	39.00 (33.00, 44.00)	53.50 (38.25,61.00)	59.00 (51.00, 65.00)	60.00 (55.00, 68.00)	< 0.001
Male (n, %)	11 (44.00)	29 (72.50)	60 (70.59)	58 (64.44)	0.071
Smoking (n, %)	3 (12.00)	9 (22.50)	32 (37.65)	23 (25.56)	0.046
Duration of T2DM (years)	-	–	10.00 (5.00, 15.00)	15.00 (10.00, 20.00)	< 0.001^b^
BMI (kg/m^2^)	22.00 (20.55, 22.75)	23.43 (22.24,26.29)	24.21 (22.66, 26.28)	24.77 (22.78, 27.12)	< 0.001
Hypertension (n, %)	0 (0.00)	17 (42.50)	46 (54.12)	79 (87.78)	< 0.001
HbA1c (%)	-	–	8.55 (7.70, 9.90)	8.27 (7.04, 9.74)	0.110^b^
Hb (g/L)	138.56 ± 12.73	133.80 ± 16.80	142.25 ± 14.93	124.20 ± 21.29	< 0.001
Albumin (g/L)	45.80 (43.55, 47.15)	35.60 (27.93, 39.98)	40.30 (38.30, 43.30)	36.85 (33.18, 39.48)	< 0.001
HDL (mmol/L)	1.43 (1.23, 1.65)	1.21 (0.97, 1.50)	1.16 (0.98, 1.51)	1.02 (0.85, 1.36)	< 0.001
LDL (mmol/L)	2.85 (2.64, 3.35)	2.98 (2.07, 3.35)	2.40 (1.39, 3.16)	2.28 (1.51, 3.00)	0.002
Uric acid (μmol/L)	313.80 (264.65, 349.65)	325.10 (277.50, 378.25)	311.00 (250.00, 381.00)	347.50 (294.95, 411.00)	0.021
Urea (mmol/L)	4.60 (4.20, 5.70)	6.90 (5.83, 8.65)	5.80 (4.70, 6.65)	8.10 (6.38, 10.43)	< 0.001
Serum creatinine (μmol/L)	61.60 (51.70, 77.80)	97.45 (71.55, 124.23)	63.60 (53.35, 73.05)	100.55 (77.18, 142.65)	< 0.001
eGFR (mL/min/1.73m^2^)	112.20 (106.00, 117.90)	74.80 (51.85, 105.67)	103.00 (96.65, 111.25)	66.55 (44.33, 91.33)	< 0.001
UACR (mg/g UCr)	-	1070.21 (206.47, 2017.53)	9.02 (6.46, 12.57)	765.55 (116.51, 2301.56)	< 0.001^a^
Urinary NAG (U/g UCr)	-	2.82 (1.33, 5.03)	5.51 (2.74, 8.14)	12.38 (7.47, 18.25)	< 0.001^a^
Urinary NGAL (μg/g UCr)	-	25.67 (10.78, 64.58)	20.05 (10.56, 29.52)	29.66 (17.20, 58.64)	0.001^a^
Diabetic retinopathy (n, %)	-	–	26 (30.59)	54 (60.00)	< 0.001^b^
Use of RASi (n, %)	0 (0.00)	34 (85.00)	31 (36.47)	67 (74.44)	< 0.001
Use of SGLT2i (n, %)	0 (0.00)	14 (35.00)	39 (45.88)	49 (54.44)	< 0.001

^a^comparisons between the non-DKD group, the T2DM-DKD group, and the T2DM+DKD group.

^b^comparisons between the T2DM-DKD group and the T2DM+DKD group.

BMI, body mass index; DKD, diabetic kidney disease; eGFR, estimated glomerular filtration rate; Hb, hemoglobin; HbA1c, hemoglobin A1c; HDL, high-density lipoprotein cholesterol; LDL, low-density lipoprotein cholesterol; NAG, N-acetyl-β-D-glucosaminidase; NGAL, neutrophil gelatinase-associated lipocalin; RASi, renin-angiotensin system inhibitors; SGLT2i, sodium-glucose co-transporter 2 inhibitors; T2DM, type 2 diabetes mellitus; UACR, urinary albumin-to-creatinine ratio; UCr, urinary creatinine.

### 3.2 Urinary succinate levels in different study groups and in subgroups of patients with DKD

There were no statistically significant differences in log_10_-transformed urinary succinate levels between healthy controls and patients with non-DKD. However, patients with T2DM without DKD presented significantly higher log_10_-transformed urinary succinate levels than healthy controls (*p* < 0.05). Notably, patients with DKD exhibited the highest log_10_-transformed urinary succinate levels among all four groups (all *p* < 0.001) (**Figure 1A**). Subgroup analyses were performed to investigate whether urinary succinate levels reflect the severity of kidney injury in DKD. Patients with DKD and a UACR ≥ 300 mg/g Ucr had significantly higher log_10_-transformed urinary succinate levels than those with a UACR < 300 mg/g Ucr (*p* < 0.01) (**Figure 1B**). Similarly, patients with DKD and an eGFR < 60 mL/min/1.73m² had higher log10-transformed urinary succinate levels than those with an eGFR ≥ 60 mL/min/1.73 m² (*p* < 0.001) (**Figure 1C**).

**Figure 1:**
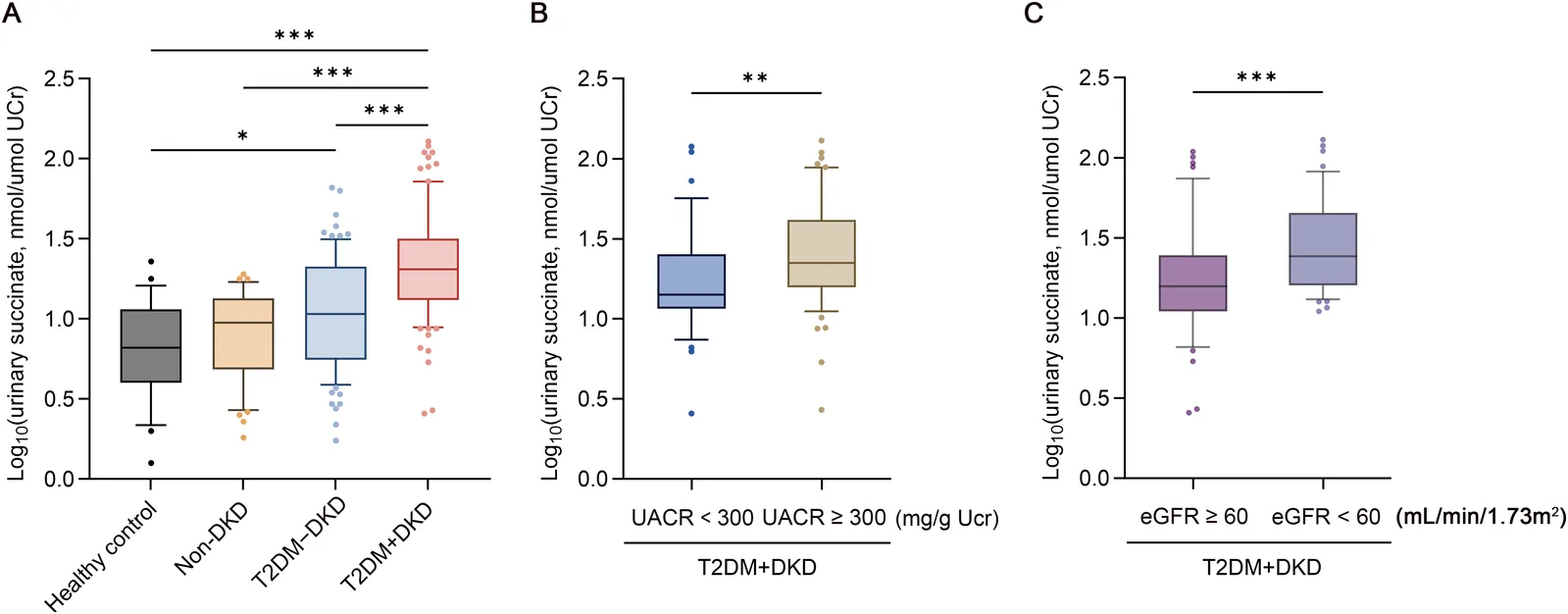
Urinary succinate levels in different study groups and in subgroups of patients with DKD. **(A)** Log_10_-transformed urinary succinate levels in healthy controls (n = 25), patients with non-DKD (n = 40), patients with T2DM without DKD (n = 85) and patients with DKD (n = 90) group. **(B)** Log_10_-transformed urinary succinate levels in patients with DKD and UACR < 300 mg/g Ucr (n = 35) or ≥ 300 mg/g Ucr (n = 55). **(C)** Log_10_-transformed urinary succinate levels in patients with DKD and subgroups of patients with eGFR < 60 mL/min/1.73m² (n = 42) or ≥ 60 mL/min/1.73m² (n = 48). The boxplot shows the distribution of the data by displaying the median, quartiles, and range. *p < 0.05, **p < 0.01, ***p < 0.001 as indicated. eGFR, estimated glomerular filtration rate; DKD, diabetic kidney disease; T2DM, type 2 diabetes mellitus; UACR, urinary albumin-to-creatinine ratio; UCr, urinary creatinine.

### 3.3 Characteristics of patients with T2DM stratified by median level of urinary succinate

Patients with T2DM were stratified into low- and high-urinary succinate groups based on the median level of log_10_-transformed urinary succinate. As shown in **Table 2**, patients with higher urinary succinate levels were older, had a longer duration of T2DM, and exhibited a higher prevalence of comorbid hypertension and diabetic retinopathy (all *p* < 0.05). Notably, patients in the high urinary succinate group showed more severe kidney injury, as indicated by significantly lower eGFR and higher serum creatinine, UACR, NAG, and NGAL levels (all *p* < 0.05). Additionally, these patients showed decreased Hb, albumin, and HDL (all *p* < 0.05). No significant differences were found in terms of gender distribution, smoking history, BMI, HbA1c, LDL, uric acid, urea, or the use of RASi and SGLT2i between the two groups (all *p* > 0.05).

**Table 2:** Characteristics of patients with T2DM stratified by median level of urinary succinate.

Characteristics	Low urinary succinate [log_10_(urinary succinate) < 1.18] (n = 87)	High urinary succinate [log_10_(urinary succinate) ≥ 1.18] (n = 88)	*p*
Age (years)	59.00 (50.00, 65.00)	61.50 (55.25, 69.00)	0.005
Male (n, %)	64 (73.65)	54 (61.36)	0.085
Smoking (n, %)	29 (33.33)	26 (29.55)	0.589
Duration of T2DM (years)	10.00 (5.00, 17.00)	14.00 (10.00, 20.00)	0.002
BMI (kg/m^2^)	24.73 (22.85, 27.07)	24.35 (22.17, 26.89)	0.207
Hypertension (n, %)	53 (60.92)	72 (81.82)	0.002
HbA1c (%)	8.09 (7.37, 9.45)	8.60 (7.34, 9.79)	0.578
Hb (g/L)	140.18 ± 17.38	125.83 ± 20.98	< 0.001
Albumin (g/L)	39.30 (37.60, 42.10)	38.00 (34.50, 40.55)	0.002
HDL (mmol/L)	1.16 (0.95, 1.72)	1.02 (0.88, 1.24)	0.003
LDL (mmol/L)	2.49 (1.30, 3.07)	2.27 (1.52, 3.03)	0.892
Uric acid (μmol/L)	333.03 ± 96.89	341.16 ± 89.25	0.564
Urea (mmol/L)	6.50 (5.30, 8.10)	7.30 (5.45, 9.88)	0.065
Serum creatinine (μmol/L)	70.20 (59.60, 89.00)	83.95 (63.73, 130.75)	0.001
eGFR (mL/min/1.73m^2^)	101.10 (81.80, 110.50)	83.15 (48.08, 101.45)	< 0.001
UACR (mg/g UCr)	14.57 (7.39, 145.49)	197.29 (10.67, 1873.12)	< 0.001
Urinary NAG (U/g UCr)	5.60 (3.57, 7.85)	12.41 (8.13, 18.11)	< 0.001
Urinary NGAL (μg/g UCr)	21.61 (10.66, 33.33)	28.47 (15.97, 62.27)	0.002
Diabetic retinopathy (n, %)	33 (37.93)	47 (53.41)	0.040
Use of RASi (n, %)	45 (51.72)	53 (60.23)	0.257
Use of SGLT2i (n, %)	47 (54.02)	41 (46.59)	0.326

BMI, body mass index; eGFR, estimated glomerular filtration rate; Hb, hemoglobin; HbA1c, hemoglobin A1c; HDL, high-density lipoprotein cholesterol; LDL, low-density lipoprotein cholesterol; NAG, N-acetyl-β-D-glucosaminidase; NGAL, neutrophil gelatinase-associated lipocalin; RASi, renin-angiotensin system inhibitors; SGLT2i, sodium-glucose co-transporter 2 inhibitors; T2DM, type 2 diabetes mellitus; UACR, urinary albumin-to-creatinine ratio; UCr, urinary creatinine.

### 3.4 Correlation analysis between urinary succinate and kidney injury markers in patients with T2DM

As shown in **Figure 2**, Pearson or Spearman correlation analysis was conducted to assess the correlation between log_10_-transformed urinary succinate levels and kidney injury markers in all patients with T2DM and in subgroups stratified by DKD status. In all patients with T2DM (**Figures 2A–E**), log_10_-transformed urinary succinate levels demonstrated a significant negative correlation with eGFR (r = -0.46, *p* < 0.001) and significant positive correlations with serum creatinine (r = 0.33, *p* < 0.001), UACR (r = 0.37, *p* < 0.001), urinary NAG (r = 0.56, *p* < 0.001), and urinary NGAL (r = 0.28, *p* < 0.001). In patients with T2DM without DKD (**Figures 2F–J**), log_10_-transformed urinary succinate levels exhibited a significant positive correlation with urinary NAG (r = 0.33, *p* = 0.002). However, no correlation was observed with serum creatinine, eGFR, UACR, or urinary NGAL. In contrast, patients with DKD (**Figures 2K–O**) exhibited a negative correlation between log_10_-transformed urinary succinate levels and eGFR (r = -0.41, *p* = 0.002), as well as positive correlations with serum creatinine (r = 0.33, *p* < 0.001), UACR (r = 0.29, *p* = 0.006), urinary NAG (r = 0.52, *p* < 0.001), and urinary NGAL (r = 0.35, *p* < 0.001).

**Figure 2:**
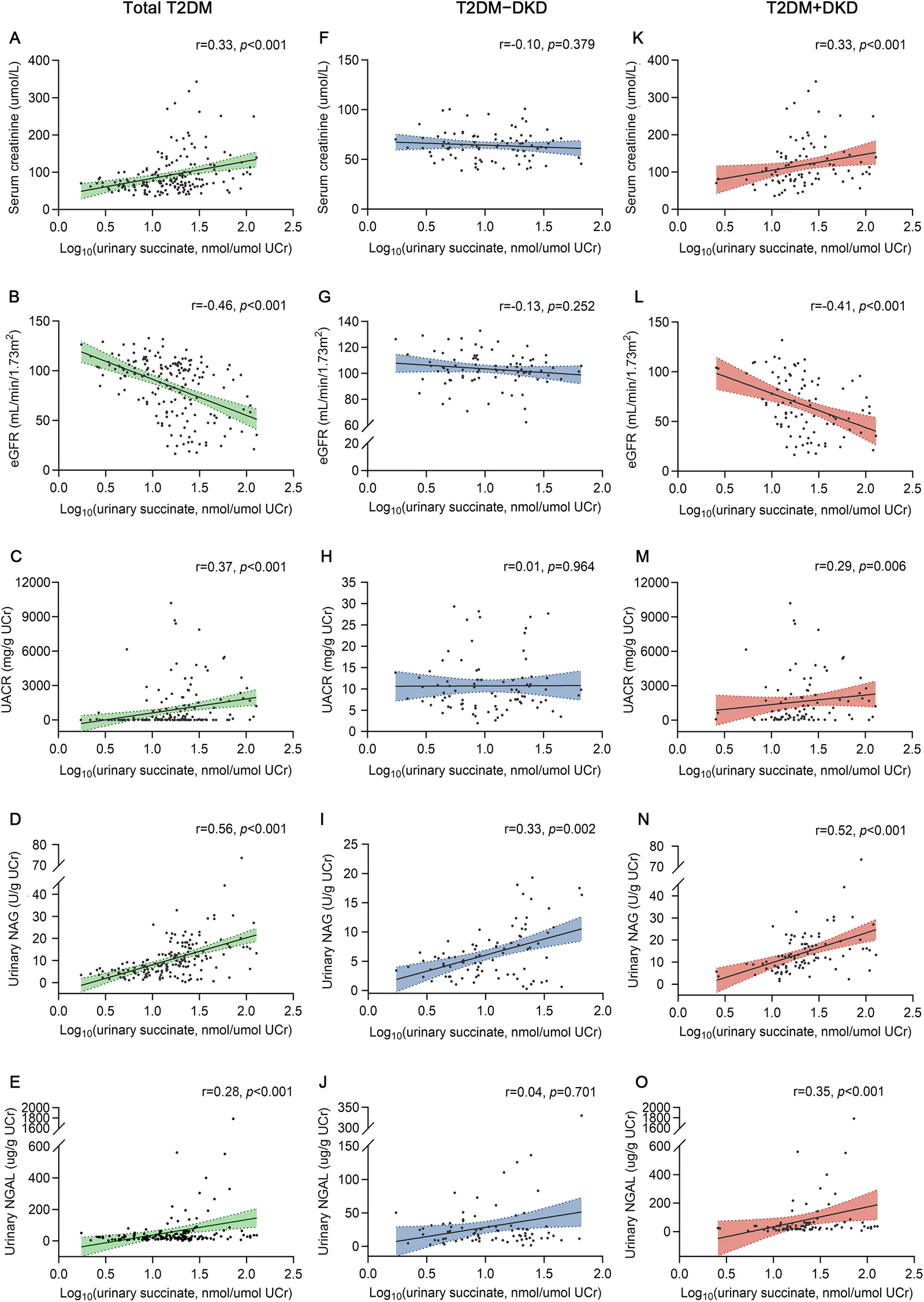
Correlation analysis between urinary succinate and kidney injury markers in patients with T2DM. Scatter plots showing correlations between log_10_-transformed urinary succinate levels and kidney injury markers in all patients with T2DM (left column), patients with T2DM without DKD (middle column), and patients with DKD (right column). Each row represents one specific kidney injury marker: serum creatinine **(A, F, K)**, eGFR **(B, G, L)**, UACR **(C, H, M)**, urinary NAG **(D, I, N)**, and urinary NGAL **(E, J, O)**. Solid lines denote linear trend lines, and shaded regions indicate 95% confidence intervals for the trend. DKD, diabetic kidney disease; eGFR, estimated glomerular filtration rate; NAG, N-acetyl-beta-D-glucosaminidase; NGAL, neutrophil gelatinase-associated lipocalin; T2DM, type 2 diabetes mellitus; UACR, urinary albumin-to-creatinine ratio; UCr, urinary creatinine.

### 3.5 Linear regression analysis for urinary succinate and eGFR in patients with DKD

Multivariable linear regression models were constructed to clarify the independent association between urinary succinate and eGFR in patients with DKD (**Table 3**). In Model 1, after adjusting for age and male sex, log_10_-transformed urinary succinate levels demonstrated a significant association with eGFR (β = -32.50, 95% CI: -49.70 to -15.30, *p* < 0.001). This association remained robust after further adjustment for conventional risk and protective factors for renal function in patients with DKD, including smoking history, hypertension, HbA1c > 7.0%, uric acid, UACR, and the use of RASi and SGLT2i (Model 2, β = -28.66, 95% CI: -46.15 to -11.71, *p* = 0.002).

**Table 3:** Linear regression analysis for urinary succinate and eGFR in patients with DKD.

Variable	Model 1			Model 2		
	β	95% confidence interval	*p*	β	95% confidence interval	*p*
log_10_(urinary succinate)	-32.50	-49.70 to -15.30	< 0.001	-28.66	-46.15 to -11.71	0.002

Model 1: adjust for age and male sex.

Model 2: adjust for age, male sex, smoking history, hypertension, HbA1c > 7.0%, uric acid, UACR, and the use of RASi and SGLT2i.

eGFR, estimated glomerular filtration rate.

### 3.6 ROC curve analysis of urinary succinate for detection of eGFR < 60 mL/min/1.73m² in patients with DKD

We performed ROC curve analysis to evaluate the diagnostic performance of urinary succinate levels for identifying eGFR < 60 mL/min/1.73m² in patients with DKD. The log_10_-transformed urinary succinate levels yielded an AUC of 0.70 (95% CI: 0.59–0.81). The optimal cutoff value of log_10_-transformed urinary succinate levels was determined to be 1.36, with a corresponding sensitivity of 57.14% and specificity of 75.00% (**Figure 3**).

**Figure 3:**
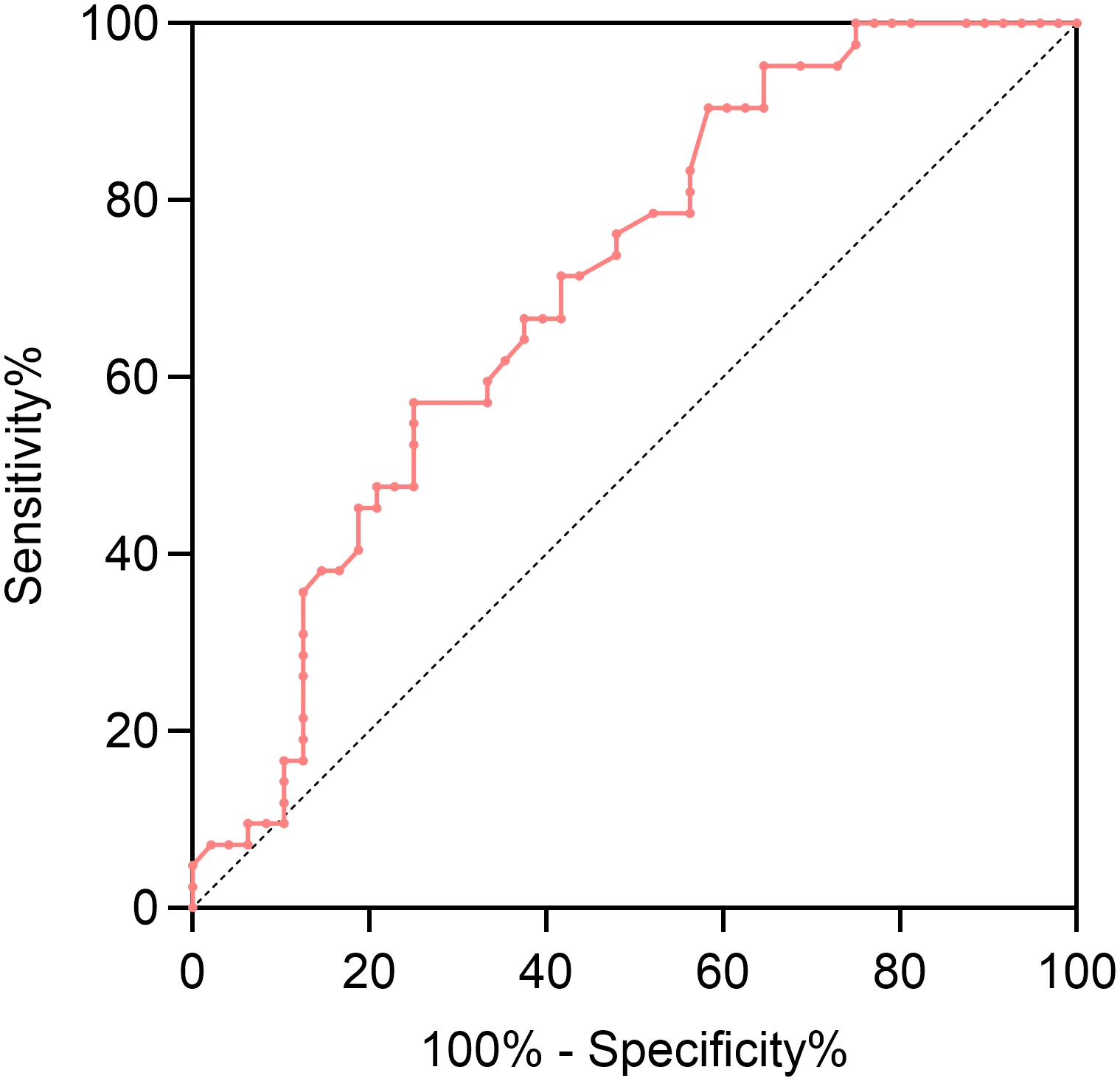
ROC curve analysis of urinary succinate for detection of eGFR < 60 mL/min/1.73m² in patients with DKD.

## 4 Discussion

In this study, we found that urinary succinate levels were significantly elevated in patients with T2DM, reaching the highest levels among those with DKD. Urinary succinate was independently associated with eGFR in patients with DKD after adjustment for conventional risk and protective factors. Finally, urinary succinate demonstrated moderate diagnostic performance for detecting eGFR < 60 mL/min/1.73m² in patients with DKD. Collectively, these findings suggest that urinary succinate may be a promising non-invasive biomarker for kidney injury in patients with diabetes.

A key highlight of our study is the inclusion of patients with non-DKD. Elevated urinary succinate levels were observed in patients with T2DM, with the highest levels found in those with DKD, whereas no comparable elevation was observed in subjects with non-DKD (**Figure 1A**). Healthy control subjects in our study were younger than patients with diabetes, as they did not have acute or chronic complications. However, no correlation was observed between age and log_10_-transformed urinary succinate levels in this group (r = 0.071, *p* = 0.623). Mechanistically, the accumulation of succinate occurs under conditions of imbalanced energy demand and the oxygen supply, which is a hallmark of diabetes and hypoxia (14, 18–20). However, this metabolic reprogramming does not appear to be a universal feature of all kidney diseases. The levels of urinary succinate in diabetic populations remain controversial due to the reported inconsistent results. A recent study revealed that patients with DKD exhibited elevated urinary succinate levels when compared with healthy controls and diabetic patients without DKD (15). No difference was observed between healthy controls and diabetic patients without DKD. In contrast, Sharma et al. (21) reported decreased urinary succinate levels in patients with T2DM and chronic kidney disease (CKD). However, this difference was no longer evident when patients with type 1 diabetes mellitus (T1DM) and CKD were included. The observed discrepancies may be attributed to variations in population composition, ethnic background, and diabetes subtype and duration across studies. Further research is necessary to validate these findings. Notably, the enrollment of patients with non-DKD as a disease control group provides a valuable reference for evaluating alterations in urinary succinate in the setting of diabetes. Our data suggest that urinary succinate elevation may be preferentially observed in diabetes-related kidney injury rather than a non-specific marker of general kidney injury. However, these findings should be interpreted with caution due to the relatively small size and clinical heterogeneity of the non-DKD subgroup.

Given the elevation of urinary succinate in T2DM, where does the increased urinary succinate originate from? Circulating succinate can be filtered through the glomerulus, and plasma succinate levels are elevated in T2DM (22). Nevertheless, urinary and renal succinate concentrations are substantially higher than circulating succinate levels in animal studies (14, 23), indicating that urinary succinate may primarily reflects intrarenal accumulation rather than passive filtration from circulation. In our preliminary unpublished observations, we found upregulated succinate abundance in renal tubular epithelial cells and cell supernatant after high-glucose stimulation, which supports the hypothesis. Whether impaired tubular reabsorption also contributes remains unknown. Since we did not measure paired plasma succinate in the current study, future studies with paired plasma and urine measurements are needed to confirm the origin of urinary succinate in DKD.

Correlation analysis showed different patterns between diabetic patients with or without DKD in our study (**Figure 2**). In patients with T2DM without DKD, urinary succinate was only correlated with NAG, showing a modest positive relationship (r = 0.33). In contrast, urinary succinate showed broader correlations with both tubular and glomerular injury markers in established DKD, with the strongest association observed for urinary NAG (r = 0.52), followed by significant correlations with eGFR (r = -0.41), NGAL (r = 0.35), and serum creatinine (r = 0.33). Tubular injury has been recognized as an early pathological event driving disease progression in DKD (24, 25). To our knowledge, few human studies have described the correlation between urinary succinate and tubular injury markers (NAG and NGAL) in patients with T2DM. Our results, showing elevated urinary succinate even in diabetic patients without overt albuminuria or eGFR decline, suggest that urinary succinate may reflect a broader spectrum of kidney injury relative to conventional tubular injury markers. The correlations between urinary succinate and eGFR, as well as UACR, were also confirmed in patients with progressive DKD (26). These findings are consistent with our observation that patients with DKD and elevated levels of urinary succinate exhibited more severe renal injury (**Table 2**). Recently, Jung et al. (15) reported an independent association between urinary succinate and DKD, as well as albuminuria. We extend these findings by evaluating the association between urinary succinate and renal function in DKD. We found that urinary succinate was independently associated with eGFR after adjustment for age, male sex, smoking history, hypertension, HbA1c > 7.0%, uric acid, UACR, and the use of RASi and SGLT2i (**Table 3**). The β coefficient of -28.66 indicates a clinically meaningful eGFR reduction associated with elevated log_10_-transformed urinary succinate levels. The persistence of this association after adjustment for UACR suggests that urinary succinate captures albuminuria-independent pathways of kidney damage in DKD. Further adjusting for the use of RASi and SGLT2i underscores the clinical relevance of urinary succinate in patients receiving these guideline-recommended renoprotective medications. 

While the cross-sectional design prevents causal inference, the observed correlation between urinary succinate and kidney injury appears to be biologically plausible. He et al. first identified succinate as an endogenous ligand for GPR91 (27), a receptor that is highly expressed in the kidney (28). A subsequent study reported that succinate accumulation activates GPR91 signaling, thereby triggering the release of renin through both vascular (endothelial) and tubular (macula densa) sites in the juxtaglomerular apparatus, linking hyperglycemia to RAS activation (14). Pharmacological inhibition of GPR91 was proved to be protective in the prevention of kidney damage in diabetic mice (29). The succinate-GPR91 complex may also induce renal tubular cell apoptosis via the extracellular signal-regulated kinase (ERK) signaling pathway (12) and renal fibrosis via the p-Akt/p-glycogen synthase kinase 3β (GSK3β)/β-catenin signaling pathway (13). Accordingly, elevated urinary succinate may serve as a surrogate marker which could help identify patients who may benefit from GPR91-targeted therapies currently under preclinical development in the future. Wang et al. (19) explained the potential mechanism independent of GPR91, showing that overproduction of succinate caused by increased peroxisomal β-oxidation of dicarboxylic acids suppresses mitochondrial fatty acid oxidation and promotes the generation of reactive oxygen species, leading to oxidative stress and glomerular injury. In multiple non-diabetic disorders, succinate accumulation has also been demonstrated to be detrimental, through the induction of inflammation and epigenetic regulation (30–33), mechanisms that may also be pathogenic in DKD. Notably, no significant correlation between HbA1c and urinary succinate was observed in patients with T2DM (r = -0.05, *p* = 0.519). Since HbA1c is a marker that reflects recent glycemic control, we hypothesize that increased urinary succinate may be associated with epigenetic reprogramming caused by prior hyperglycemic exposure, which is known as “metabolic memory” (34, 35).

From a diagnostic perspective, we provide a novel finding of urinary succinate: it yielded an AUC of 0.70 for discriminating eGFR < 60 mL/min/1.73m² in patients with DKD (**Figure 3**). Since an AUC of 0.70 is modest, urinary succinate should not be considered as a standalone diagnostic marker, but rather a potentially useful adjunct to the existing clinical parameters such as UACR and eGFR for the assessment of renal involvement in DKD. Meanwhile, it may help to detect tubular damage or renal impairment in diabetic cases with normoalbuminuria or ambiguous albuminuria, even before eGFR decline, providing a critical time window for renoprotective interventions.

There are some limitations for the current study. First, we cannot determine the causal relationships or temporal sequence between the levels of urinary succinate and kidney injury due to the cross-sectional design. Second, the association between urinary succinate and renal pathological damage was not analyzed due to the limited renal biopsy cases. Third, patients with T2DM concurrent with non-DKD were not enrolled, further studies with renal biopsy confirmation are needed to validate the levels of urinary succinate in these patients. Fourth, although major confounding factors were adjusted in regression analyses, residual confounding from unmeasured variables, including dietary succinate intake, physical activity and medication adherence, cannot be fully excluded.

## 5 Conclusion

In conclusion, urinary succinate levels are significantly elevated in patients with T2DM, particularly in those with DKD, but no such elevation was observed in patients with non-DKD. Urinary succinate correlates with markers of both tubular and glomerular injury and remains independently associated with eGFR in patients with DKD. It exhibits moderate diagnostic performance for eGFR < 60 mL/min/1.73m². Our results suggest that urinary succinate may be a promising non-invasive adjunct biomarker for assessing renal involvement in DKD. Due to the single-center cross-sectional design, large-scale, prospective, multicenter studies are required to validate our findings and further determine whether urinary succinate holds value for the early identification and risk stratification of DKD progression.

## Author’s note

An abstract of a portion of this work has been accepted for poster presentation at ASN Kidney Week 2026.

## Data Availability

The original contributions presented in the study are included in the article/supplementary material. Further inquiries can be directed to the corresponding authors.
